# Treatment of Full Eyelid Ptosis Following Botox Injection: A Case Report

**DOI:** 10.7759/cureus.55970

**Published:** 2024-03-11

**Authors:** Isam J Musharbash, Rosalie J Chakra

**Affiliations:** 1 Dentistry, Dr. Isam Musharbash Clinic, Amman, JOR; 2 Dentistry, Dr. Rosalie Chakra Clinic, Irbid, JOR; 3 Endodontics, Dr. Rosalie Chakra Clinic, Irbid, JOR

**Keywords:** complete recovery, gradual improvement, treatment, botox injections, eyelid ptosis

## Abstract

We report the case of a 24-year-old woman who was referred to one of the authors' clinics after six days of botulinum toxin type A injection to treat dynamic lines on her forehead, glabella complex, and crow’s feet area. Her first esthetic injection was done by another colleague elsewhere in a different clinic. Her main complaint was full eyelid ptosis, which started four days after her treatment and continued to aggravate until the time she sought our help. We treated it with another dose of botulinum toxins.

The patient started to notice a gradual improvement in her eyelid five days after our injection, so on day 14th, her eyelid was very closely back to normal opening; complete recovery was achieved.

Ptosis due to botulinum toxin injection was surprisingly and successfully reversed in our article by a second dose of botulinum toxins. This suggests that this management successfully treats such cases and can deliver a beneficial reversal option for practitioners and their patients. The case report concludes that ptosis should be promptly evaluated and treated through a multimodal approach.

## Introduction

In recent years, botulinum toxins have evolved from being a poison to becoming a versatile clinical tool. In the world of cosmetic enhancements, botulinum toxin injections have become very popular due to their ability to smooth wrinkles and rejuvenate the face. They are also widely used nowadays for the treatment of different types of headaches (i.e., migraine, tension, and chronic daily headaches) [1].

However, these treatments are not without potential complications. One such complication is eyelid ptosis or eyelid droopiness, a condition where the eyelid droops or falls, impacting not only appearance but also, in severe cases, impeding vision [2].

Ptosis of the upper eyelid is a common complaint following botulinum treatment of the glabellar complex, and its incidence in the literature is around 3%. It can occur from day 2 to day 10 after the injection. [3] This results from the unintentional involvement of the levator palpebrae superioris muscle, which is mainly responsible for lifting the eyelid. When neurotoxins spread beyond the intended treatment area, they can temporarily paralyze this muscle, leading to eyelid ptosis [4]. This can be treated with alpha-adrenergic drops (e.g., 0.5% apraclonidine ophthalmic solution) that will lift the upper eyelid by 1 to 2 mm due to Muller’s muscle contraction. [5] In this article, we report on the treatment, with a second injection of botulinum toxins, of a patient with unilateral eyelid ptosis following a botulinum toxin type A injection made to treat dynamic lines in the forehead, glabellar complex, and crow’s feet area.

## Case presentation

**Figure 1:**
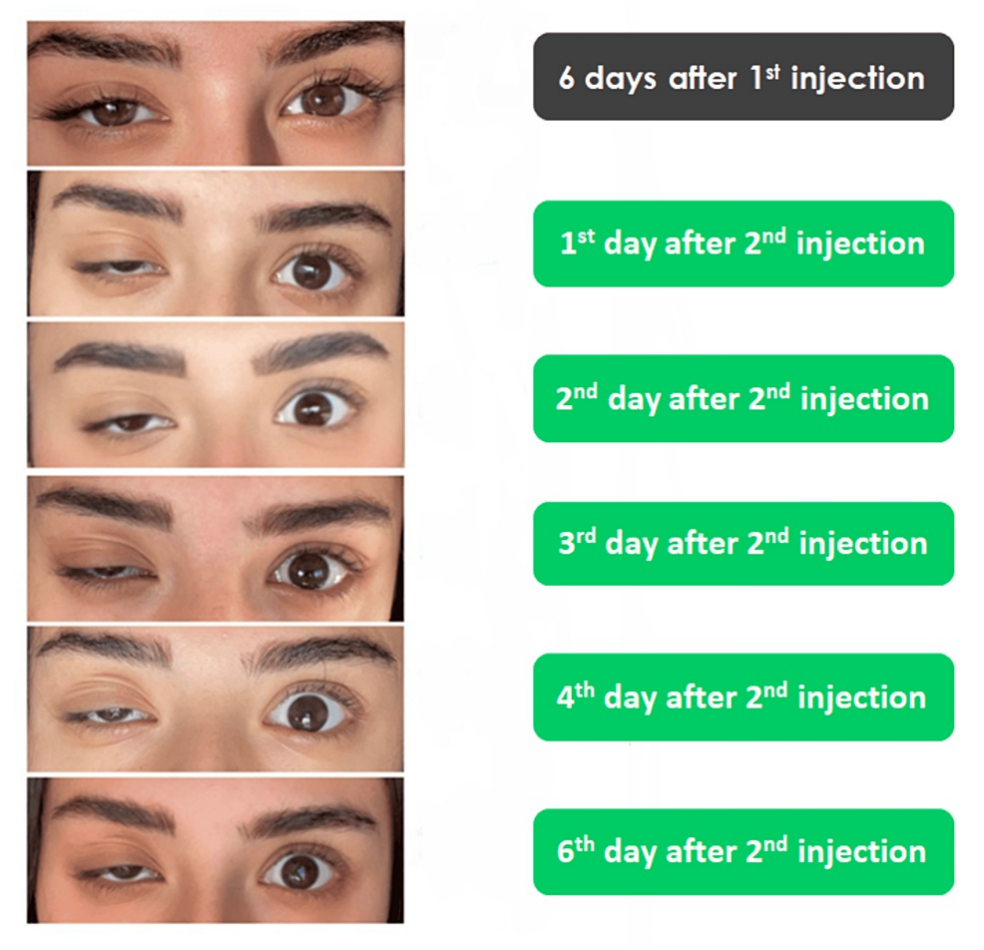
Initial situation and the progress within the first six days post-op

**Figure 2:**
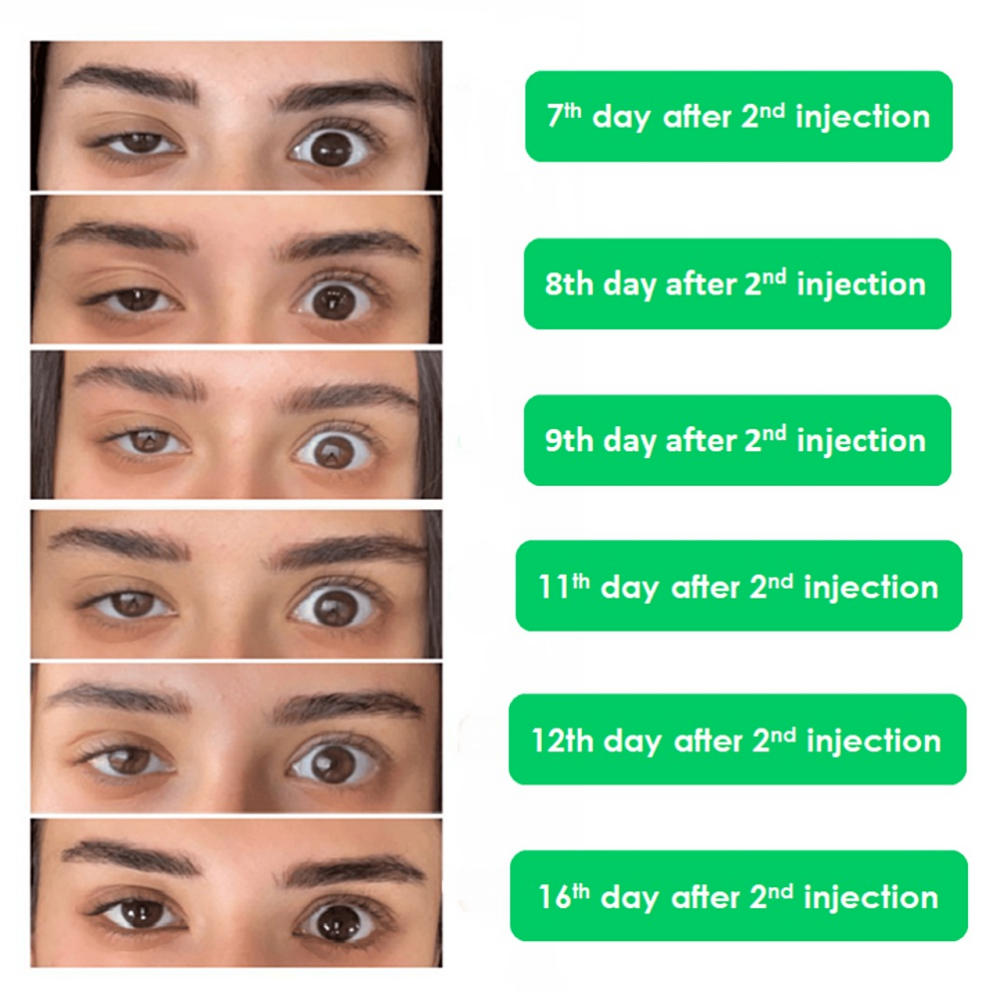
Improvement of the case from day 7 to 16 post-op

**Figure 3:**
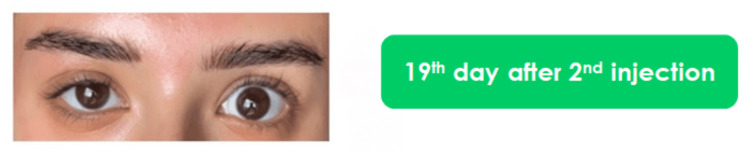
Final follow-up

A 24-year-old woman was referred to one of the authors’ clinics six days after receiving her first botulinum toxin type A injections to treat dynamic lines on her forehead, glabella complex, and crow’s feet area, elsewhere in a different clinic. Her main complaint was full eyelid ptosis in her right eye, which started four days after her treatment and continued to aggravate until the time she sought our help. Our clinic follows a protocol to treat eyelid ptosis (Table 1). The patient was initially scared to take further injections; therefore, our first management protocol at the time of her presentation to one of the author’s clinics was giving her α-adrenergic drops (i.e., 0.15% brimonidine tartrate ophthalmic solution) one droplet every eight hours to meet the patient’s preferences. However, her situation continued to worsen until we noticed that her upper right eyelid was approximately fully closed on the eighth day following her initial injection, so we decided to inject her upper eyelid in the pre-tarsal area with 2 units of BoNT/A (Botox; Allergan, Irvine, CA, USA). We used a reconstituted solution of 100 units in 2.5 ml of saline. The patient noticed a gradual improvement in her eyelid five days after our injection (Figure 1). Closely complete recovery was achieved 14 days after our injection (Figure 2), and the final follow-up was obtained after 19 days (Figure 3).

**Table 1:** Dr. Musharbash and Dr. Chakra protocol to treat eyelid ptosis

Eyelid ptosis grades (mm)	Treatment protocol
Grade 1-0 to 2 mm of eyelid ptosis	0.5% apraclonidine or 0.15% brimonidine tartrate ophthalmic solution: one droplet three times a day
Grade 2-2 to 4 mm of eyelid ptosis	Upper eyelid injection of botulinum toxins
Grade 3-4 mm to full eyelid ptosis	Upper eyelid injection of botulinum toxins and 0.5% apraclonidine or 0.15% brimonidine tartrate ophthalmic solution: one droplet from one to three times a day according to patients’ preference (they may put one drop before leaving to work if they are employed and omit the night dose since they are at home)

## Discussion

There are a lot of structures through which the facial anti-wrinkle injections can pass. These include the supraorbital foramen (hole), a small groove at the superior and medial margins of the orbit in the frontal bone [6]. Several practitioner factors contribute to minimizing the risk of eyelid ptosis. This includes injecting superficially the corrugator's lateral tail and brow area, especially the medial injection, due to the ease of leak and the proximity to the levator palpebrae superioris muscle. Moreover, injecting only three frown injections, excluding those in the lateral tail of the corrugator, will minimize the risk of eyelid ptosis [7]. Further, post-injection pressure application (i.e., gentle pressure on the glabella area post-injection) from bottom to top for about 30 seconds will minimize the diffusion of botulinum toxins inside the orbit. This helps localize the neurotoxin’s effect and reduce its migration [8].

Patients also play a role in prevention. They should be advised to avoid manipulating the treated area for a certain period after receiving injections, as rubbing or massaging can cause the toxin to spread unintentionally [9]. By integrating these preventative strategies, including the specific technique of applying pressure post-injection, minimal effective dosing, and a three-point injection technique, practitioners can significantly diminish the risk of causing eyelid ptosis. Ptosis of the upper eyelid is a common complaint after botulinum treatment, as are debilitating postoperative complications that may impair patients’ satisfaction and quality of life. To our knowledge, a few botulinum-induced eyelid ptosis cases treated with botulinum injections were reported before the present report.

The eyelid elevator muscles are the levator palpebrae superioris and superior tarsal. These muscles are innervated with different nerves. The oculomotor nerve innervates the levator palpebrae superioris (i.e., a voluntary muscle) [10], while sympathetic nerves innervate the superior tarsal (Müller) muscle (i.e., a smooth muscle) [11]. The superior tarsal muscle is approximately 12 mm long and originates on the undersurface of the levator superioris. This muscle inserts superiorly on the tarsal border and elevates the upper lid approximately by 2 mm. Alpha-adrenergic receptor agonists (e.g., apraclonidine or brimonidine tartrate) reverse the ptosis by directly stimulating the sympathetic innervations of the superior tarsal muscle. This could open the eyelid by 1 to 2 mm. The effect of these eye drops is known to last for six to eight hours. However, patients with botulinum-induced eyelid ptosis may suffer for several weeks until the effects of the toxin wear off [12].

The eyelid, like most moving structures in the body, has muscles that oppose each other. Eyelid retractors, the tarsal, and the levator palpebrae muscles are opposed by the palpebral part of orbicularis oculi, the protractors [13-14] Therefore, if the palpebral part of orbicularis oculi, anterior to the tarsal muscle, is injected, then we can lift a drooping eyelid by a millimeter or so [6,7]. Based on the fundamental knowledge of anatomy, muscle function, activity, and the mechanism of action of botulinum toxin type A, the authors of this article propose a protocol to treat eyelid ptosis.

## Conclusions

Ptosis does occur after elective botulinum treatment. Disproportionate postoperative symptoms raising suspicion of ptosis should be promptly evaluated and treated through a multimodal approach.

This case report recommends that patients be directed to play a role in prevention. They should be advised to avoid manipulating the treated area for a certain period after receiving injections, as rubbing or massaging can cause the toxin to spread unintentionally.
